# The first report on the sortase-mediated display of bioactive protein A from *Staphylococcus aureus* (SpA) on the surface of the vegetative form of *Bacillus subtilis*

**DOI:** 10.1186/s12934-021-01701-4

**Published:** 2021-11-17

**Authors:** Samira Ghaedmohammadi, Gholamreza Ahmadian

**Affiliations:** 1 Department of Cellular and Molecular Biology, Estahban Higher Education Center, Estahban, Iran; 2 Department of Industrial and Environmental Biotechnology, National Institute of Genetic Engineering and Biotechnology (NIGEB), Tehran, Iran

**Keywords:** Surface display, *Bacillus subtilis*, Protein A

## Abstract

Protein A (SpA) is one of the most important *Staphylococcus aureus* cell wall proteins. It includes five immunoglobulin (Ig)-binding domains which can bind to immune complexes through the Fc region of immunoglobulins. The binding of SpA to the polymeric supports can be used to prepare affinity chromatography resins, which are useful for immunoprecipitation (IP) of antibodies. Protein A is also used to purify many anti-cancer antibodies. In this study, SpA was displayed on the surface of *Bacillus subtilis* cells using a sortase-mediated system to display the target protein to the *B. subtilis* cell wall. A series of plasmids consisting of cassettes for cell wall-directed protein A as well as negative controls were constructed and transformed into *B. subtilis* WASD (*wprA sigD*) cells. SDS-PAGE, western blot, flow cytometry, functional IgG purification assay, and a modified ELISA assay were used to confirm the surface display of SpA and evaluate its function. Semi-quantitative ELISA results showed that the binding capacity of lyophilized *Bs*-SpA is 100 μg IgG from rabbit serum per 1 mg of cells under optimal experimental conditions. Low production costs, optimal performance, and the use of a harmless strain compared to a similar commercial product predict the possible use of SpA immobilization technology in the future.

**Supplementary Information:**

The online version contains supplementary material available at 10.1186/s12934-021-01701-4.

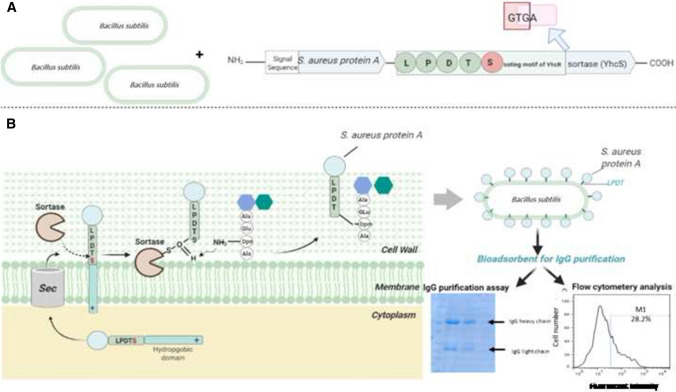

## Background

The expression of recombinant proteins fused with signal peptides to sort and direct them to different parts of the bacterial cell surface is called surface display which has many applications such as peptide libraries screening, whole-cell bioconversion, live vaccine production, and whole-cell metal adsorbents [20].

The surface display is a suitable alternative for immobilizing recombinant proteins and enzymes on various polymeric supports and living cells because, unlike traditional physical and chemical immobilization approaches that require various steps, it is biocompatible and not time-consuming or challenging to achieve [25].

There are several ways to efficiently display heterologous polypeptides on the surface of the bacterial cells. Various surface proteins such as outer membrane lipoproteins including LamB, OmpA, and OmpF in gram-negative bacteria are used as fusion partners to express bacterial and viral antigens [3, 8, 22, 37]. Several strategies have also been used to express heterologous proteins on the surface of the vegetative form of gram-positive bacteria or their spores [7, 13]. Gram-positive bacteria are more rigid because they have a much thicker peptidoglycan layer than that of gram-negative bacteria. They also lack a lipid outer membrane envelope, which simplifies the extracellular secretion of heterologous proteins [32]. In addition, some species of gram-positive bacteria are safe for human health and therefore the most desirable options for use in the surface display applications as well as other biotechnology purposes [2]. Among the gram-positive bacteria, *B. subtilis* has more attractive properties and has many industrial and biotechnological applications [19]. Its genetics and physiology have intensively been known, and among bacteria, understanding its genetic background is second only to *E. coli* [25]. One of the most attractive strategies used for superficial display of homologous and heterologous proteins by a number of gram-positive bacteria is sortase-mediated immobilization.

Gram-positive bacteria express a membrane-anchored transpeptidase enzyme called sortase A that cleaves an LPXTG sorting signal in the C-terminal region of precursor proteins destined for cell wall anchoring [33]. Many of the known surface proteins in Gram-positive bacteria are covalently immobilized by sortase on the cell wall, which have cell wall anchoring properties, including the presence of an N-terminal signal peptide and a cell wall sorting signal in the C-terminal region which is highly conserved. The sorting signal consists of a conserved penta-peptide motif, LPXTG, a hydrophobic stretch of 15–22 amino acids and a short charged tail (6–7 a.a), making up a total of approximately 35 a.a. In *S. aureus*, the LPXTG motif serves as the recognition sequence for proteolytic cleavage between the threonine and glycine residues, followed by subsequent linkage of threonine to a branched peptide, via the penta-glycine cross-bridge amino group, in the peptidoglycan layer. During export via the Sec protein translocation machinery, the sorted proteins are retained within the cytoplasmic membrane due to their C-terminal hydrophobic region and the charged tail. The Sec machinery (or translocase) is a major pathway in bacteria for protein translocation from the cytosol across the cytoplasmic membrane [27]. The use of this type of general sortase-mediated cell wall anchoring has been the most widely used strategy for display of heterologous proteins on the surface of gram-positive bacteria [32]. However, other types of carrier proteins and various other mechanisms have also been used [1].

In *B. subtilis*, there are two distinct putative sortases (YhcS and YwpE) and several putative substrates, of which YhcR and YfkN have been extensively studied and first reported by Fasehee et al. [9]. YhcS is known as the major *B. subtilis* sortase and is responsible for covalently anchoring proteins including YhcR on the cell wall [24]. The penta-peptide recognition and cleavage motifs in the C-terminal region of the YhcR, as a natural sortase substrate, is LPDTS. In 2011, Fasehee et al. displayed the chitinase of *B. pumilus* on the surface of *B. subtilis* using protein engineering and the YhcS sortase system by fusing the sorting signal of the YhcR to the carboxyl-terminal region of the chitinase, thus proving the functionality of the YhcS [9].

Protein A from *S. aureus* (SpA) is one of the most extensively studied cell wall proteins with a molecular weight of 40–60 kDa. Its molecular weight depends on the numbers of immunoglobulin (Ig)-binding domains in the N-terminal region [14, 34]. The C-terminal part has no Ig-binding domain and contains the sorting signal to covalently binds to the peptidoglycan cross bridge [26]. This region is composed of LPXTG cleavage motif, a hydrophobic stretch of amino acids followed by a positively charged tail which are necessary for interaction and attachment of the protein to the *S. aureus* cell wall. The Ig-binding region includes five small three-helix-bundle domains (E–D–A–B–C) separated by conserved flexible linkers that share up to 90% amino acid sequence identity with each other [5, 36]. SpA is a valuable component used in methods such as agglutination and ELISA assay as well as western blotting in molecular biology laboratories. It can also bind to immune complexes and immunoglobulins in blood and serum, which has been widely used in biotechnology. Immobilized SpA on polymeric supports can be used as affinity chromatography resins for immunoprecipitation (IP) of antibodies from biological solutions [11, 18, 29, 30, 39].

SpA technology, especially immobilized SpA, has the advantages of interacting with the Fc region of Ig molecule without interrupting its ability to bind to antigens, leading to the development of several applications in immunological research.

The aim of this study was to introduce the structural gene of SpA along with the YhcS sortase into *B. subtilis* as a host for display of SpA on bacterial cell surface and the development of a bioadsorbent. It would provide a safer means than a similar commercial product that uses *S. aureus* for Ig immunoprecipitation by natural immobilization of SpA on the cell surface. In this experiment, for the first time, the native *B. subtilis* sortase, YhcS, as well as the sorting signal derived from its native substrate, YhcR, were used to display *S. aureus* protein A.

## Results

### Expression vectors for surface display

Different constructs including pNC1 and pNC2 were constructed as negative control plasmids and pSpA-YhcS as a main plasmid for SpA surface display (Table 1). The plasmid constructs were first transformed in *E. coli* as a cloning host and then into *B. subtilis* as an expression host (Additional file 1: Fig. S3, S4). The constructed recombinant *B. subtilis* strains were called (*Bs*-NC1, *Bs*-NC2) as negative control strains and *Bs*-SpA as a main strain for SpA surface display. *Bs*-NC1 is a recombinant *B. subtilis* transformed with pNC1 [a recombinant pHY plasmid containing spa gene and cell wall sorting motif (CWM) but lacks the enzyme sortase]. *Bs*-NC2 is a recombinant *B. subtilis* transformed with pNC2 (a recombinant pHY plasmid containing spa gene but lacks the enzyme sortase and CWM). *Bs*-pHY is a recombinant *B. subtilis* transformed with pHY300-PLK and *Bs*-SpA is a recombinant *B. subtilis* transformed with pSpA-YhcS [a recombinant pHY plasmid containing spa gene, CWM and *B.subtilis* sortase (YhcS)]. More details about plasmids will be discussed in the “Methods” Section.

**Table 1:** Plasmids and strains

Plasmids and strains	Relevant properties	References
Plasmids		
pHY300-PLK	*B. subtilis* protein expression vector; Ap^r^, TC^r^	NIGEB collection
pChiM	Recombinant pHY plasmid containing Chitinase (ChiS), cell wall sorting motif and sortase (YhcS)	Previous study
pSpA-YhcS	Recombinant pHY plasmid containing spa gene, cell wall sorting motif (CWM) and *B.subtilis* sortase (YhcS)	This study
pNC1	Recombinant pHY plasmid containing spa gene and cell wall sorting motif but lacks the enzyme sortase	This study
pNC2	Recombinant pHY plasmid containing spa gene but lacks the enzyme sortase and CWM	This study
Strains		
*E.coli* (*DH5α*)	F- endA1 glnV44 thi-1 recA1 relA1 gyrA96 deoR nupG Φ80d*lacZ*ΔM15 Δ(*lacZYA-argF*)U169, hsdR17(rK − mK +), λ −	Invitrogen
*B. subtilis* (WASD)	trpC wprA::kan sigD::cat	NIGEB collection
*Bs-pHY*	*B. subtilis* (WASD) transformed with pHY300-PLK	This study
*Bs-SpA*	*B. subtilis* (WASD) transformed with pSpA-YhcS	This study
*Bs-NC1*	*B. subtilis* (WASD) transformed with pNC1	This study
*Bs-NC2*	*B. subtilis* (WASD) transformed with pNC2	This study

*B. subtilis* WASD cells were used in all experiments. WASD is a strain of *B. subtilis* that is knocked out for two surface proteases [cell wall binding protease (wprA) and sigma D (sigD)] for more efficient surface display of heterologous proteins [21].

### Extraction of cell wall immobilized proteins using lysozyme treatment, SDS-PAGE and western blot analysis

**Fig. 1:**
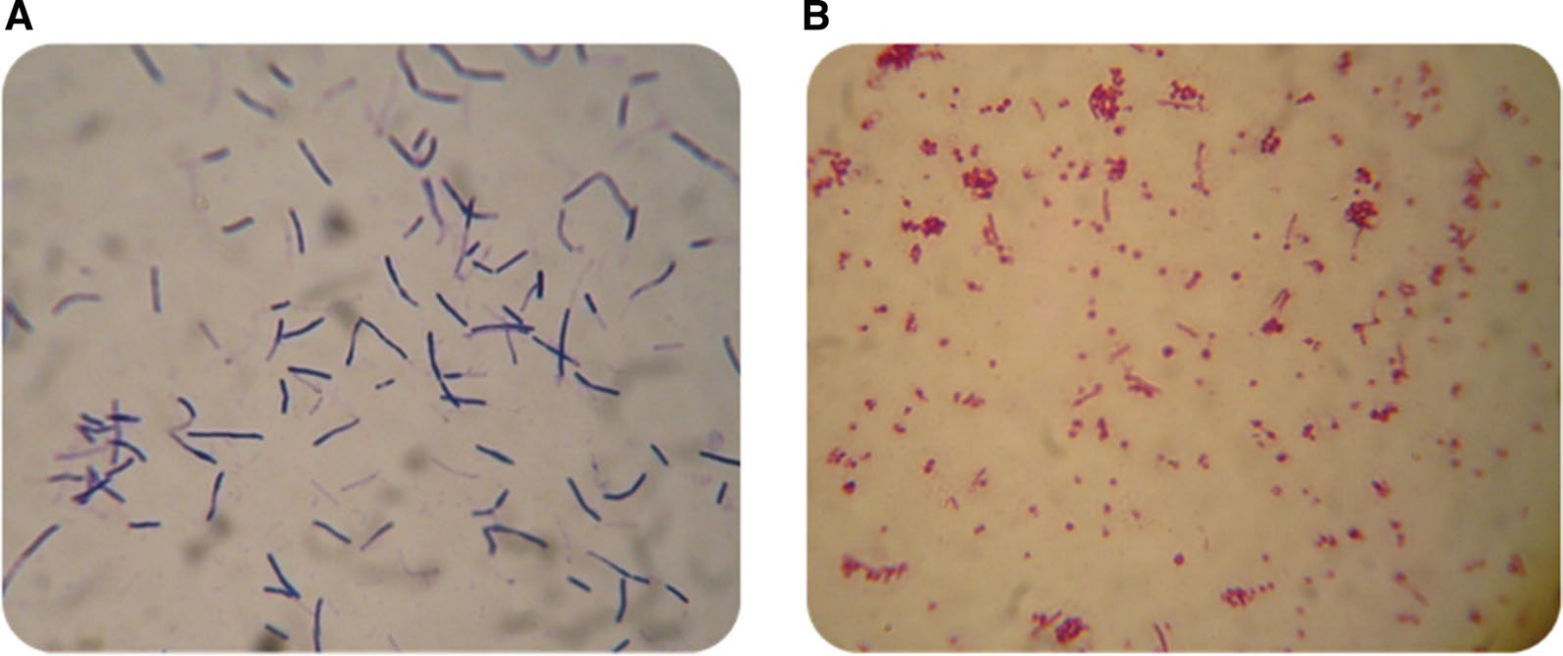
Confirmation of cell wall separation from protoplast using gram staining. **A** *B. subtilis* cells before lysozyme treatment and **B** *B. subtilis* cells after lysozyme treatment and cell wall separation

A standard method to verify the proper separation of the cell wall from protoplasts and extracting its cell wall-anchored proteins, after treatment with a lysosome, is gram staining. As can be seen in the Fig. 1, the bacteria lose their rod shape and become spherical after separating the cell walls, because the bacterial peptidoglycan preserves the specific shape of each bacterial species. This indicates that the cell wall layer that maintained the bacterial rod shapes has been removed. In addition, prior to treatment with lysosome, the cells have a thick layer of peptidoglycan in the cell wall that retains the dye, crystal violet. After treatment with lysosome and removal of the peptidoglycan, the crystal violet will be washed out on addition of alcohol, and the cells turn red after addition of safranin.

**Fig. 2:**
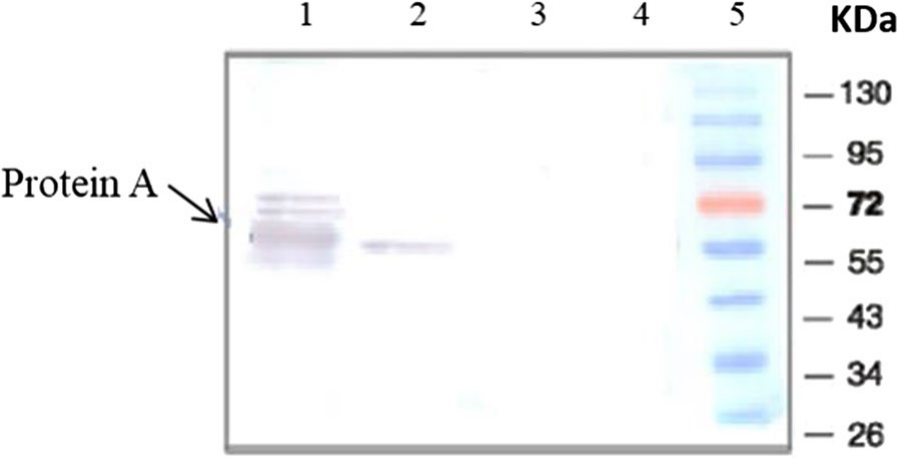
Confirmation of the presence of Protein A on the surface of the *B. subtilis* cells using western blot analysis of protein extracted from the cell wall fractions. Lane 1: cell walls fraction from *Bs*-SpA; Lane 2: cell wall fraction from *Bs*-NC1; Lane 3: cell wall fraction from *Bs*-NC2; Lane 4: cell wall fraction from *Bs-*pHY. Lane 5: protein marker

Protein fractions from the cell wall and protoplast of the recombinant *B. subtilis* WASD including negative controls were evaluated by SDS-PAGE and western blot analysis to determine their Protein A content. In order for cell wall proteins not to be contaminated with the cytoplasmic and protoplasmic proteins and to be able to prove that the protein of interest (SpA) is immobilized on the cell wall (compared to controls), we have to treat the cells with lysozyme to gently shave and separate the cell wall-anchored fraction from protoplast proteins. As proteins are first translated in the cytoplasm and then are transported out of the cell through the Sec secretory pathway, a small percentage of these proteins are also found inside the cytoplasm. As shown in Fig. 2, Protein A of about 50 kDa was observed in the cell wall samples isolated from surface-engineered *Bs*-SpA and also in a very small amounts on the surface of *Bs*-NC1cells. Protein A was found mostly in the protoplasts of the negative control bacteria.

Negative control plasmid construct pNC1 lacks the native *B. subtilis* sortase, YhcS, but has the LPDTS sorting motif that have been shown in our previous publication [9] that is identified by YhcS. Although *Bs*-NC1 strain has been transformed with the plasmid that lacks the overexpressed YhcS, the native endogenous sortase is able to identify this motif and immobilize a small amount of protein A onto the cell surface. However, the amount of surface-stabilized protein A by this control strain is lower than that of the *Bs*-SpA strain in which YhcS is overexpressed.

Further confirmation of this result, seen in the lane 3 and 4 for the *Bs*-NC2 strain and the *Bs*-pHY strain, respectively. These strains lack the sortase cleavage and recognition motif and therefore the protein A is not immobilized on their surface.

**Fig. 3:**
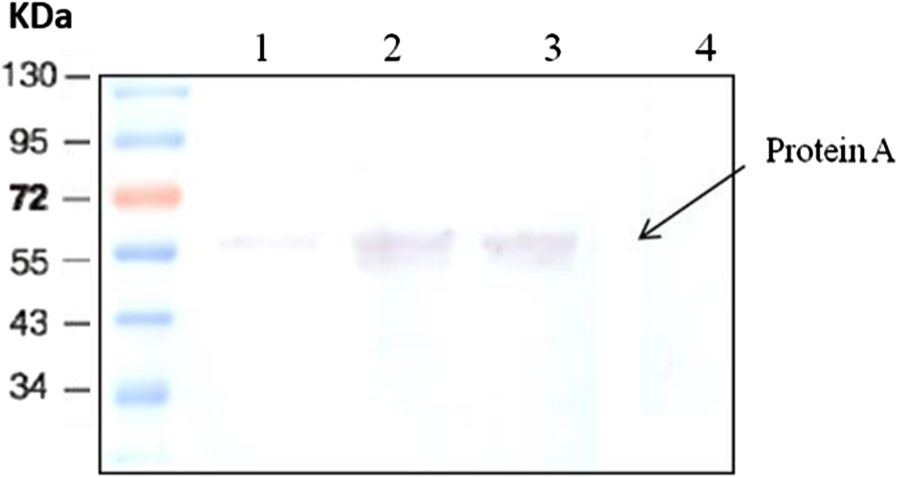
Western blot analysis using protoplast samples isolated from various *B. subtilis* strains developed in this study. Lane 1: protoplasts fraction from *Bs*-SpA; Lane 2: protoplasts fraction from *Bs*-NC1; Lane 3: protoplast fraction from *Bs*-NC2; Lane 4: protoplast fraction from *Bs*-pHY

On the other hand, western blot analysis was also performed on proteins extracted from protoplast of different bacterial strains, the results of which are shown in Fig. 3. Lane 1 in Fig. 3, corresponding to proteins extracted from protoplasts of the *Bs*-SpA, shows a weak band which indicates a small amount of protein A in its protoplast fraction.

However, in protein samples extracted from the protoplast of *Bs-NC1* and *Bs-NC2* strains, higher amounts of protein A are present in protoplast samples, indicating that proteins synthesized within the cytoplasm cannot be transported to the cell surface and are accumulated in the cytoplasm. These results demonstrate the efficiency of the developed surface display system in this study and the efficiency of sortase YhcS used in recombinant *Bs-*SpA. The *Bs*-pHY control strain, as expected, does not express any protein A in its cytoplasm.

### Flow cytometry analysis of SpA display on the surface of *B. subtilis* strains

**Fig. 4:**
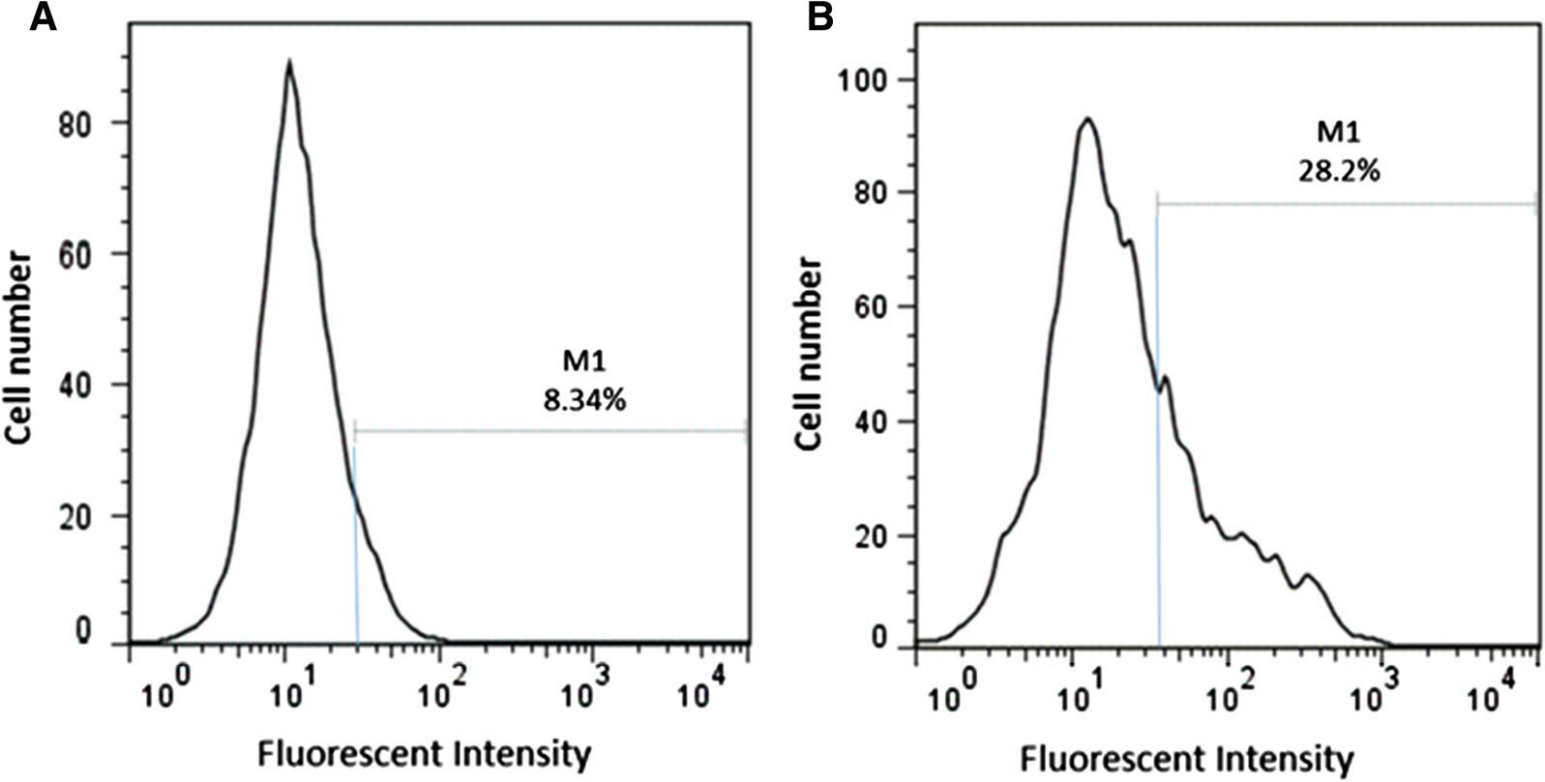
Flow cytometry analysis, in the FACS format, of surface modified and negative control recombinant *B. subtilis* cells. **a** *Bs*-NC1 strain as a negative control, **b** *Bs-*SpA strain. Vertical line denotes the number of cells and horizontal line is the strength of fluorescence. M1 shows a gate to detect only cells that fall in a positive area. All the cells outside this gate are ignored as a background. The cells that are located in M1 region represent the cell that display the SpA protein on their surface

Quantification of the SpA expression on the surface of the recombinant *B. subtilis* cells was evaluated by flow cytometry. The results showed that the percentage of fluorescent *Bs*-NC1 strains, which was used as a negative control, and *Bs-SpA* strains, which showed in above-mentioned experiments to expressed the highest amount of the protein A on the cell surface, were about 8% and 28%, respectively. Significant increase in fluorescence in the *Bs-*SpA strains compared to the negative control strain is another confirmation of the efficiency of this surface display system and the expression of protein A on the surface of the developed strain (Fig. 4).

### Whole cell SpA assay

**Fig. 5:**
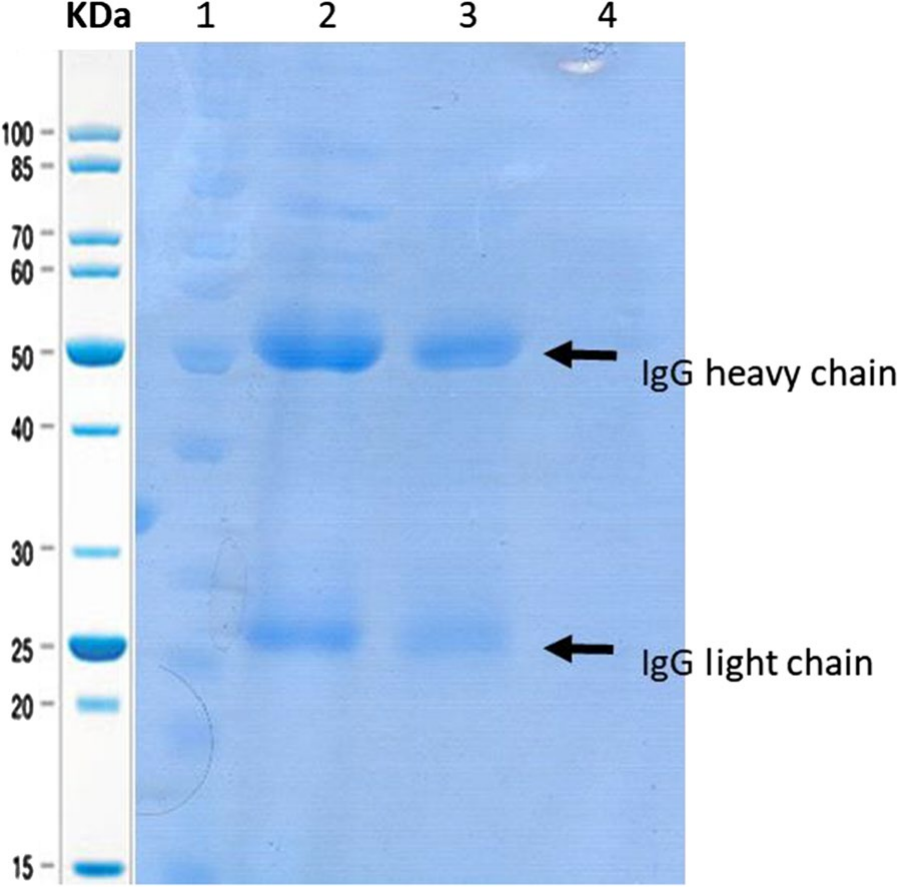
Purification of rabbit serum IgG by recombinant *B. subtilis* strains. IgG heavy chain is about 50 KDa and IgG light chain is about 23 KDa. Lane 1. Protein marker; Lane 2. Supernatant from serum incubation with the commercial protein A-agarose as a positive control; Lane 3. Supernatant from incubation of serum with the recombinant *Bs-SpA* strain; Lane 4. Supernatant from serum incubation with the recombinant *Bs*-pHY strain as a negative control

The functional analysis of protein A on the surface of different *B. subtilis* strains were evaluated by their ability to bind IgG and immunoprecipitate them from rabbit serum. The result showed that the surface-modified bacteria displaying protein A are able to purify the IgG from other serum proteins just like commercial protein A-agarose beads (Fig. 5).

The Experiments were carried out with 5 and 10 mg of the lyophilized recombinant bacterial strains and according to test results and considering the ease of use and cost reduction, 5 mg of freeze-dried strains was chosen. ELISA results showed that the binding capacity of lyophilized *Bs*-SpA is 100 μg rabbit IgG per 1 mg of cells under the optimal experimental conditions.

The amount of IgG purified by the recombinant strain was approximately 55% of the IgG amount purified by the commercial protein A-agarose in the functional assay using equal amount of both strain and agarose beads in terms of weight.

Preparation of protein A-agarose as a commercial product includes the preparation of beads, activation of its surface functional groups, and on the other hand, expression and purification of the recombinant protein A, and then the final step of protein A immobilization on the surface of agarose beads in a covalent form. Considering the various steps and problems of preparing protein A-agarose, as well as the lower cost of using the recombinant strain and the simplicity of its preparation for antibody purification, and cost-effectiveness, justifies its lower efficiency in antibody purification efficiency compared to protein A-agarose.

### Reusability of *Bs*-SpA

**Fig. 6:**
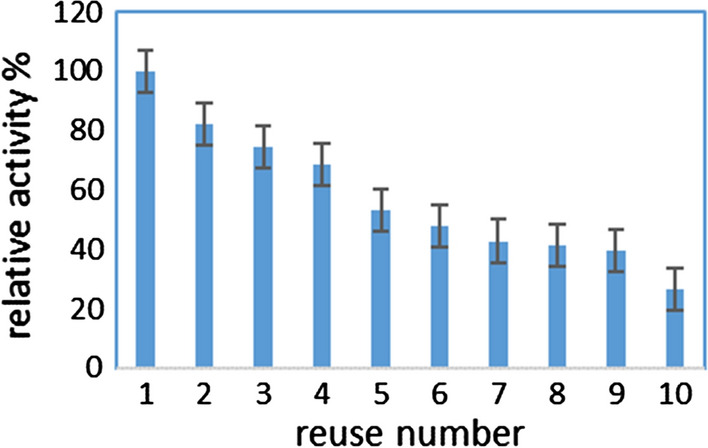
The reusability of *Bs*-SpA. After 6 uses, the SpA activity was reduced to about a half and after 10 times uses, it was reduced to a quarter

Matrix recycling and its reusability for many times is one of the most important advantage of protein surface display in industry. Therefore, the expenses regarding protein expression, purification, and immobilization can be decreased. After elution of the bound IgG from the *Bs*-SpA, they were re-used to bind the IgG in the rabbit serum several times. The amount of antibody bound to the *Bs*-SpA was evaluated for each round of IgG binding/elution, and the activity was compared with the initial activity, which was assumed as 100%. The residual activity of the immobilized protein A was found to decrease after multiple uses. As shown in the Fig. 6, after 6 uses, about 50% of the IgG-binding activity is still preserved.

## Discussion

Protein A has five Ig binding domains through which can bind to the Fc region of antibodies. This property is used in the pharmaceutical and biotechnology industries for the purification of antibodies with therapeutic applications as well as the purification of antigen–antibody complexes by immunoprecipitation and then proteomic analysis. This property of SpA has been used as immobilized on the polymeric surfaces as well as on various surfaces of living cells by surface display.

The first use of *S. aureus* cells carrying protein A as an IgG-binding agent was demonstrated by Jonsson et al. [15] which provided a process for quantifying Alpha-fetoprotein (AFP) tumor marker in human serum. Kessler then published two papers on the benefits of protein A-displaying staphylococci as a bioadsorbent for IgG in the immunoprecipitation process [16, 17].

In 1994, Djojonegoro et al. developed a filamentous bacteriophage M13-based display system for a single Ig-binding domain of the *S. aureus* Protein A*.* By expressing the B domain of protein A on the bacteriophage surface, the phage was able to interact with the immobilized IgG molecules that could be used to purify phage [6].

In the present study, unlike our previous study, which was based on *B. subtilis* spores surface display, the vegetative form of the *B. subtilis* were used as a safe vehicle to display the *S. aureus* protein A [10]. Spore surface display system has some drawbacks, including the fact that the process of separating spores is a complex, time-consuming, and relatively expensive process, and it is also relatively difficult to obtain large amounts of pure spores.

On the other hand, the surface display system developed based on the vegetative form of the *B. subtilis* has not been studied as much as the spore display system [19] and few studies have been reported in which the system based on the sortase and sortase substrates to display heterologous proteins [24]. In particular, this vegetative surface display has been reported based on sortase of other gram-positive bacteria such as *S. aureus* and *L. monocytogenes* [28], but the vegetative surface display system based on *B. subtilis* sortase and its native substrate has not been reported, except for a report by Fasehee et al. [9].

Herein we described the display of protein A on the surface of *B. subtilis* cells using Yhcs sortase and its native substrate, YhcR. The recombinant strain of *B. subtilis* developed in this project (*Bs*-SpA) is used as a formalin-fixed and heat-killed.

The strain developed as a bioadsorbent in this study, compared to the similar commercial products developed by different companies like Merck, that uses *S. aureus* naturally displaying protein A, is a safe and probiotic strain and has many advantages over those commercial products.

The cost reduction and optimal performance of *Bs-*SpA predict the potential application of this immobilization technology in the relevant immunological and biotechnological research. This product is routinely used for IgG purification and immunoprecipitation assays.

In this study, we did not use the *S. aureus* sortase because it is not functional in the *B. subtilis* host as the cross-bridge of lipid II precursors differs in structure between bacterial species. In *B. subtilis* and *L. monocytogenes*, the cross-bridge amino group is derived from the side chain of m-diaminopimelic acid (m-Dpm). In contrast, the *S. aureus* cross-bridge contains five glycine residues that are tethered to the 1-amino group of lysine within lipid II [C55-(PO3)2-MurNac(L-Ala-D-iGln-Lys(NH2-Gly5)-D-Ala-D-Ala)-GlcNAc]. Sortase A enzymes of different bacterial species have evolved to recognize cross-bridge structures of that species [33].

## Conclusions

Display of proteins and peptides on microorganisms (bacteria, yeast, and phages) is becoming a leading technology due to low cost and its various applications. The non-pathogenicity of *B. subtilis* as a safe surface make this technology applicable to food and biological industries. Also, the advantages of easy purification and recycling of immobilized enzyme can greatly reduce the cost of industrialization. The surface-engineered, formalin-fixed and heat-killed strain of *Bs*-SpA prepared in this study, expresses protein A on the surface of its vegetative form and is able to bind and purify immunoglobulins and can be a safe alternative to the biological products on the market offered by companies. In this research, Yhcs sortase system was used to display protein A on the surface of *B. subtilis* cells. The genetic method used in this study has other advantages, including the fact that this method eliminates the need for the complex and time-consuming steps, including the preparation and purification of resins and polymer surfaces, surface activation, protein purification and immobilization. It is also very cost-effective, especially for laboratories that perform large number of immunoprecipitation tests.

It is believed that with further research on *B. subtilis* surface display, this technology will play an important role in more fields in the future.

## Methods

### Plasmids, bacterial strains and growth conditions

The bacterial strains and plasmids used in this study are listed in Table 1. *E. coli* strain DH5α and *B. subtilis* strain WASD were used as the cloning and expression host for protein A surface display, respectively. *E. coli* cells were propagated at 37 °C on LB agar plates. For protein production, *B. subtilis* cells were cultured in superrich medium (SRM) (Bacto tryptose, 25 g/liter; yeast extract, 20 g/liter; dipotassium phosphate, 3 g/liter; glucose, 4.5 g/liter; pH 7.5) at 30 °C for 14 h and harvested for analysis.

### DNA techniques

**Fig. 7:**
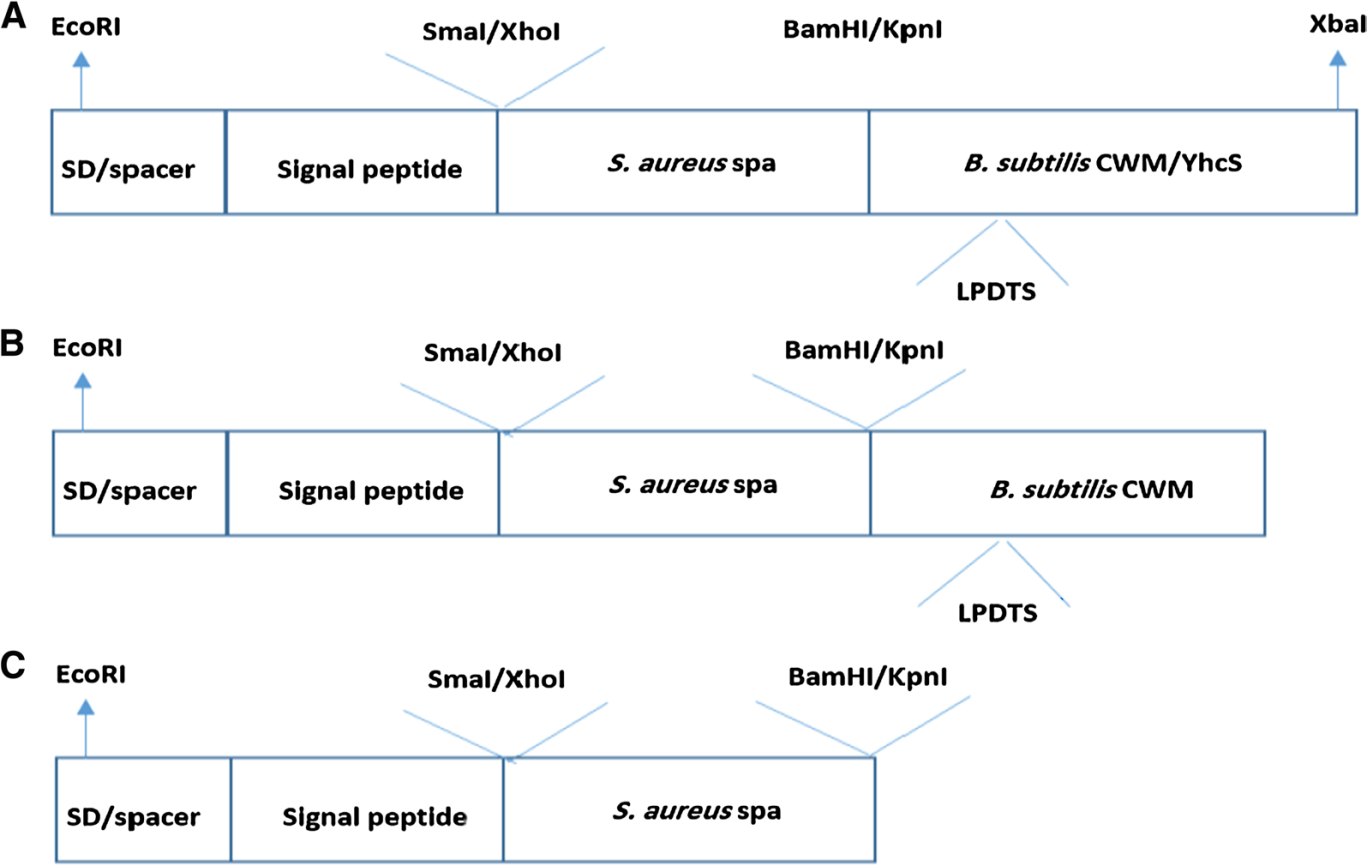
Schematic representation of SpA display constructs. Plasmids were constructed to express the SpA with or without the C-terminal cell wall motif (CWM) of YhcR, and with or without the sortase YhcS. In the genome of *B. subtilis*, the stop codon of yhcR (TGA) and the start codon of yhcS (GTG) overlap. This feature was retained in our constructs to ensure that both genes are transcribed in a similar manner from the same promoter. **a** pSpA-YhcS is the main construct for SpA display on the *B. subtilis* cell wall surface, **b** the control construct named pNC1 lacks the sortase, **c** the control construct named pNC2 lacks both the CWM and sortase. *SD* Shine Dalgarno sequence; *spa* *S. aureus* protein A; *CWM* cell wall motif; *YhcS*: sortase of *B. subtilis*

The sortase-mediated system was used for displaying protein A on the bacterial cell wall. To achieve this goal, the following constructs, including various controls, were designed in such a way that the cleavage motif and sortase were located adjacent to each other in a cassette (Fig. 7). Meanwhile, the start codon of the sortase and the stop codon of the modified substrate overlapped. It should be noted that a native signal peptide of protein A is used in these constructs, since an N-terminal signal peptide is necessary for the Sec protein translocation machinery as described in the introduction.

The negative control constructs (pNC1 and pNC2 related to Table 1) were designed using restriction enzyme digestion by enzymes cleavage sites embedded in the main construct or by designing primers and amplification by PCR (Additional file 1: Table S1 for oligonucleotide sequences).

The plasmid PHY300 was used as a shuttle vector for cloning the constructs in the* E. coli* (DH5α) and then for expression in the main host, *B. subtilis* (WASD). In this plasmid ampicillin and tetracycline are selection markers recognized by *E. coli* and *B. subtilis*, respectively. The tetracycline is also used as an expression inducer.

After ligation, the constructs were transformed into the competent *E. coli* (DH5α) cells by the thermal shock method [31]. The standard method of calcium chloride was used for this purpose. Several different methods such as plasmid extraction and enzymatic digestion, were then used to verify the recombinant clones. After confirmation of cloning, the recombinant plasmids were extracted from the primary host. *B. subtilis* transformation was performed by the Spizizen competent cells [35] and the natural transformation method. In this method, the bacteria’s culture becomes more deficient in different stages, so that *B. subtilis* forced to absorb DNA from the environment [23]. In addition to these constructs, plasmid pHY300 with no insert was transformed into the bacteria and used as another negative control (refer to the Additional file 1 for more detail about plasmids construction and strain development).

### Protein A expression on the cell wall of the recombinant *B. subtilis*

For this purpose, *Bs*-pHY, *Bs*-SpA, *Bs*-NC1, and *Bs-NC2* were grown in the SRM culture containing tetracyclin (final concentration of 25 μg/ml) at 30 °C for 14 h and 180 rpm. Since the target gene is cloned downstream of the tetracycline resistance gene in the plasmid pHY300, it is under the control of its promoter and is constitutively expressed.

### Extraction of cell wall-bound proteins using lysozyme

To confirm that the protein A is immobilized on the cell surface of *B. subtilis* and covalently linked to its cell wall, the bacterial cell wall was isolated from the protoplast as a first step according to Welbull’s article with some modification as follows and the presence of protein A was investigated in this fraction [38]. The cells from a late log-phase *B. subtilis* culture were collected by centrifugation, washed, pelleted, and finally resuspended in 150 µl protoplast buffer (20% sucrose, 50 mM Tris pH 7.5, 15 mM MgCl_2_) [pH 7.6], 0.2 mg/ml Lysozyme). Samples were incubated at 37 °C for 30 min. Protoplasts were separated from the extracted cell wall fraction by centrifugation at 5400×*g* for 7 min. The resulting protoplast pellet was resuspended in 150 µl of protoplast buffer. For checking the accuracy of the method, bacterial cells were stained using Gram’s method and were observed under the light microscope [4]. The cell wall and protoplast fractions were used for further analysis, such as ELISA and western Blotting.

### SDS-PAGE and western blot analysis

Cell wall and protoplast fractions from a specific number of obtained bacterial cells were analyzed by SDS-PAGE to determine their protein A content. Due to the limited surface loading capacity of each cell and to further confirm the surface expression of protein A, its expression was also determined by western blot analysis using a specific antibody. For western blot analysis, the proteins were transferred to polyvinylidene fluoride (PVDF) membranes and treated with a rabbit anti-goat IgG antibody bound to horseradish peroxidase (HRP) in a 1:500 dilution. The blot was developed with hydrogen peroxide and 4-chloronaphthol as a substrate.

### Evaluation of cell surface binding of SpA by flow cytometry

Quantitative determination of SpA expression on the surface of *B. subtilis* cells was examined by flow cytometry. For this purpose, the cells were washed three times with phosphate-buffered saline (PBS), resuspended in 1 mL PBS solution containing rabbit antibody conjugated to FITC (1:1000) and incubated on ice for 1 h. Cells were washed again three times with PBS, and resuspended in 500 µL of PBS. Fluorescent signals created on the surface of the control and SpA-conjugated cells were analyzed using a fluorescence-based flow cytometer (FACSCalibur, BD, USA). The Cell Quest ver. 1.0 software was used for data analysis.

### Bacterial fixation with formaldehyde

Bacterial formaldehyde fixation was performed in such a way that cell surface proteins are preserved. Briefly, after growing the bacteria, they were centrifuged, the supernatant was discarded, and the bacterial pellet was dissolved in 100 ml PBS  +  0.02 sodium azide. The pellets were then washed twice and weighed. Bacteria were dissolved in PBS 10% wt/vol and formaldehyde was slowly added to the final concentration of 1.5%. The bacterial suspensions were placed on a stirrer for 90 min at room temperature. After centrifugation, the bacterial pellets were dissolved in 10% w/w PBS and incubated at 80 °C for 5 min with gentle rotation. The bacterial suspensions were then placed in cold water and after several washing steps as before, they were kept at 4 °C until testing.

### IgG binding assay using surface-engineered bacteria

The ability of surface-modified *B. subtilis* to bind and purify IgG was analyzed by the binding assay using rabbit serum. 30 ml of the surface-modified bacteria or 10 mg of their dried powder were resuspended and washed with 1 ml of starting buffer [100 mM Tris–HCl, (pH 8)]. The pH of rabbit serum was adjusted to 7.5–8 with 1 M Tris (pH 8), and added to the bacterial prep and incubated for 1 h. Thereafter, the samples were centrifuged and washed with 100 mM Tris–HCl (pH 8) and 10 mM Tris–HCl (pH 8), respectively. Bound IgGs were eluted using a solution of 100 mM glycine (pH 3) containing 1 M KCl after which were quickly neutralized with 1 M Tris–HCl (pH 8). Bacterial samples were then washed with starting buffer and stored at 4 °C for reuse. Eluted fractions of IgG were analyzed on SDS-PAGE and quantified by ELISA. In all experiments, protein A-agarose beads supplied as a 50% slurry in 20% Ethanol from Abcam was used as the positive control.

### Protein A coating-ELISA (PAC-ELISA) test

A modified version of the ELISA called PAC-ELISA was used to measure the amount of IgG eluted from the surface-modified bacteria according to Hobbs et al. with some modification to increase the efficiency of coating antibody onto the 96-well plates [12]. Briefly, SpA was diluted in 100 mM carbonate buffer [3.03 g Na_2_CO_3_ and 6.0 g NaHCO_3_ in 1 l of distilled water (pH 9.6)] and added to plates at a final concentration of 10 μg/ml. Eluted IgG from the surface-modified *B. subtilis* and other strains used as a negative control, were added to different wells. Serial dilutions of the rabbit antibody were subsequently added as standards. After blocking with 1% (w/v) Bovine Serum Albumin (BSA) and a final washing step, an HRP-conjugated anti-rabbit antibody at 1:2000 dilution was added to each well, and the development of color reaction was carried out by adding ABTS [2,2′-Azinobis (3-ethylbenzothiazoline-6-sulfonic acid)-diammonium salt] substrate. The absorbance was determined by the ELISA reader at 405 nm. The standard curve was plotted and the concentration of purified antibodies was calculated.

### Reusability of *Bs*-SpA

*B.* subtilis cells can be easily purified and isolated from the culture by centrifugation. IgG purification from rabbit serum and its quantification was used for reusability analysis of the Bs-SpA. At the end of each cycle of reusing, the *Bs*-SpA was removed from the reaction and washed three times with 10 mM Tris buffer (pH 8), after which a sample of new serum was added to the *Bs*-SpA cells to start a new cycle. The relative activity was calculated by defining the activity of the first reaction as 100%.

## Supplementary Information

**Additional file 1: Figure S1.** pChiM is a plasmid containing cell wall sorting motifs of *B. subtilis* (CWM), sortase cleavage motif (LPDTS) and the enzyme sortase (YhcS). This plasmid was used as a template for making other constructs prepared in this study. **Figure S2.** Schematic representation of SpA display constructs. Plasmids were constructed to express the SpA with or without the C-terminal cell wall motif (CWM) of YhcR, and with or without the sortase YhcS. **a** pSpA-YhcS is the main construct for SpA display on the *B. subtilis* cell wall surface, **b** the control construct named pNC1 lacks the sortase, **c** the control construct named pNC2 lacks both the CWM and sortase. *SD* Shine Dalgarno sequence; *spa* *S. aureus* protein A; *CWM* cell wall motif; *YhcS* sortase of *B. subtilis*. **Figure S3.** Diagrammatic representation of the constructs used in this study as control plasmids (pNC1 and pNC2) and main construct (pSpA-YhcS). **Figure S4.** The process of making constructs and their transformation. **Table S1.** Oligonucleotide primers sequences used in this study.

## Data Availability

Not applicable.
