# Supplementary material for: The first report on the sortase-mediated display of bioactive protein A from Staphylococcus aureus (SpA) on the surface of the vegetative form of Bacillus subtilis
Source: Microb Cell Fact. 2021 Nov 17;20:212. doi: 10.1186/s12934-021-01701-4 (PMC8596801; doi:10.1186/s12934-021-01701-4)
Supplement: Supplementary file 1 — Additional file 1: Figure S1. pChiM is a plasmid containing cell wall sorting motifs of B. subtilis (CWM), sortase cleavage motif (LPDTS) and the enzyme sortase (YhcS). This plasmid was used as a template for making other constructs prepared in this study. Figure S2. Schematic representation of SpA display constructs. Plasmids were constructed to express the SpA with or without the C-terminal cell wall motif (CWM) of YhcR, and with or without the sortase YhcS. a pSpA-YhcS is the main construct for SpA display on the B. subtilis cell wall surface, b the control construct named pNC1 lacks the sortase, c the control construct named pNC2 lacks both the CWM and sortase. SD Shine Dalgarno sequence; spa S. aureus protein A; CWM cell wall motif; YhcS sortase of B. subtilis. Figure S3. Diagrammatic representation of the constructs used in this study as control plasmids (pNC1 and pNC2) and main construct (pSpA-YhcS). Figure S4. The process of making constructs and their transformation. Table S1. Oligonucleotide primers sequences used in this study. [file 12934_2021_1701_MOESM1_ESM.docx]

**Supplementary file**

**Construction of the recombinant strains**

The construction of the recombinant strains is completely described here for more clarification.

pChiM is a plasmid made in a previous study in our group containing cell wall sorting motifs of *Bacillus subtilis* (CWM), sortase cleavage motif (LPDTS) and the enzyme sortase (YhcS). This plasmid was used as a template for making other constructs in this study (Figure S1).

The main final construct is shown in figure S2 (below). As can be seen in figure, part of the plasmid construct (Figure S2, shown in gray) was synthesized and it was then subcloned in a *B. subtilis* shuttle vector called pHY300PLK and used as the negative control 2 construct (pNC2). The non-colored part of figure S2 was amplified by PCR using the primers PCF4 and PCR4, (listed in Table S1, below) and the plasmid pChiM as a template.

Plasmid pCN2 and the amplified non-colored part of figure S2 were then digested with the same restriction enzyme (*BamH1* and *Xba1*) and ligated to each other to form the final construct named pSpA-YhcS (Figure S2).

The negative control 1 construct (pNC1) which contains spa gene, cell wall sorting motif (CWM), and sortase cleavage site (LPDTS) but lacks the enzyme sortase was amplified by PCR using NCFG and NCRG primers (Table S1) and pSpA-YhcS as a template. The PCR product was digested with the restriction sites *SalI* and *XbaI* and were cloned in in the multiple cloning site of the plasmid pHY300PLK.

All three constructs and also pHY300-PLK (Figure S3) were first transformed into the competent *E. coli* (DH5α) cells as a cloning host and finally transformed into the *B. subtilis (WASD)* cells as a main expression host (Figure S4).


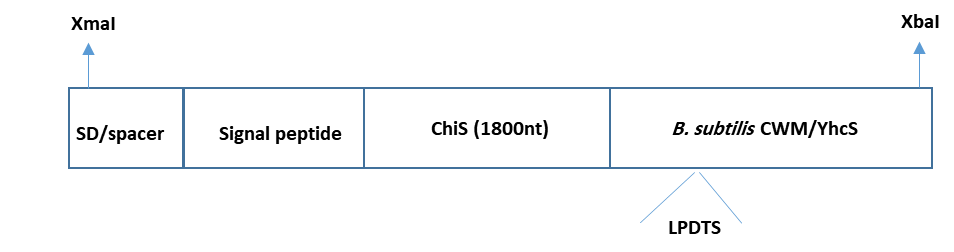


Figure S1. pChiM is a plasmid containing cell wall sorting motifs of *B. subtilis* (CWM), sortase cleavage motif (LPDTS) and the enzyme sortase (YhcS). This plasmid was used as a template for making other constructs prepared in this study.


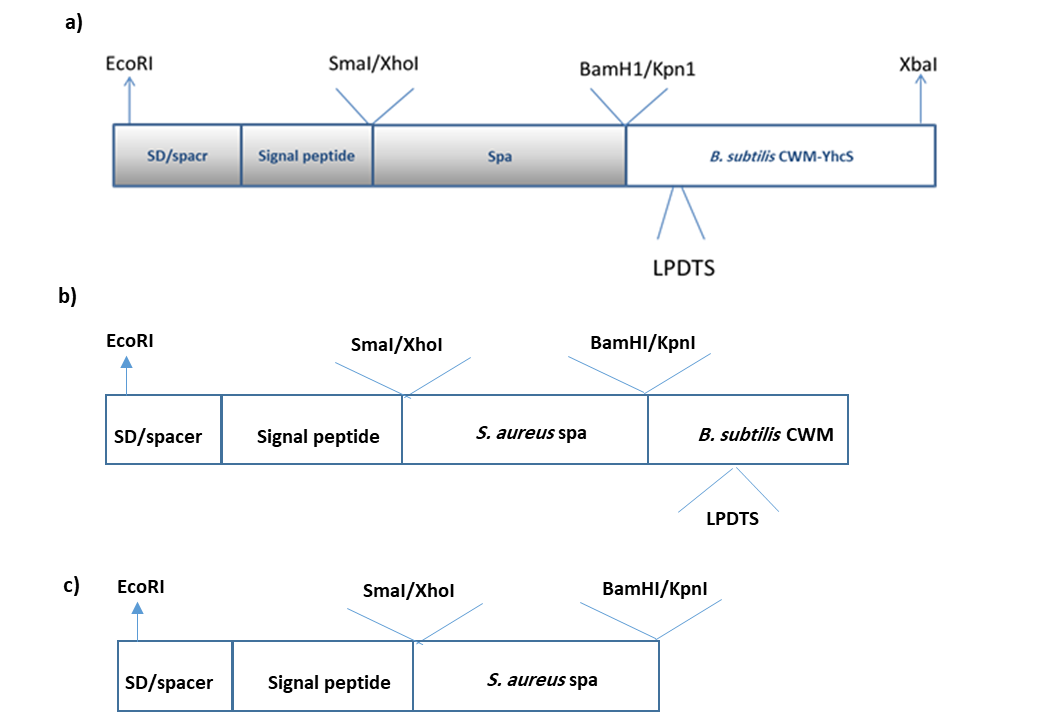


Figure S2. Schematic representation of SpA display constructs. Plasmids were constructed to express the SpA with or without the C-terminal cell wall motif (CWM) of YhcR, and with or without the sortase YhcS.. a) pSpA-YhcS is the main construct for SpA display on the *B. subtilis* cell wall surface, b) The control construct named pNC1 lacks the sortase, c) The control construct named pNC2 lacks both the CWM and sortase. SD: Shine Dalgarno sequence, spa: *S. aureus* protein A, CWM: cell wall motif, YhcS: sortase of *B. subtilis*

Table S1. Oligonucleotide primers sequences used in this study

| Primer names | Sequence | description |
| --- | --- | --- |
| PCF4 | 5'GGGGGATCCGAAAGCGGGTCTGATCATCAGC 3' | The underlined sequence is *BamH1* recognition site |
| PCR4 | 5'GGGTCTAGATTAAGTCACTCGTTTTCCATATATAATATAG 3' | The underlined sequence is *Xba1* recognition site |
| NCFG | 5' GGGGGTCGACAAAGGAGGCGATTTG 3' | The underlined sequence is *SalI* recognition site |
| NCRG | 5' GGGTCTAGACACGTTCTGGAGGCGC 3' | The underlined sequence is *Xba1* recognition site |


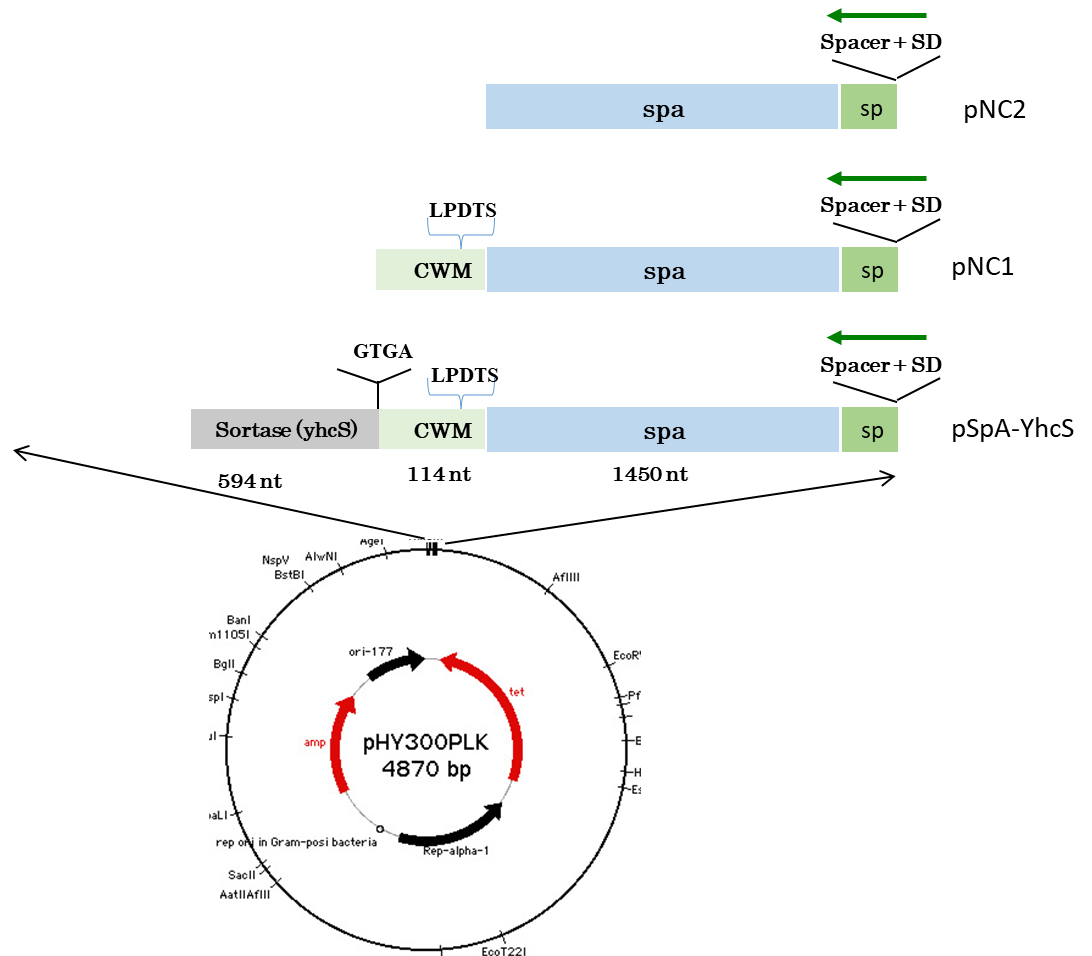


Figure S3. Diagrammatic representation of the constructs used in this study as control plasmids (pNC1 and pNC2) and main construct (pSpA-YhcS).


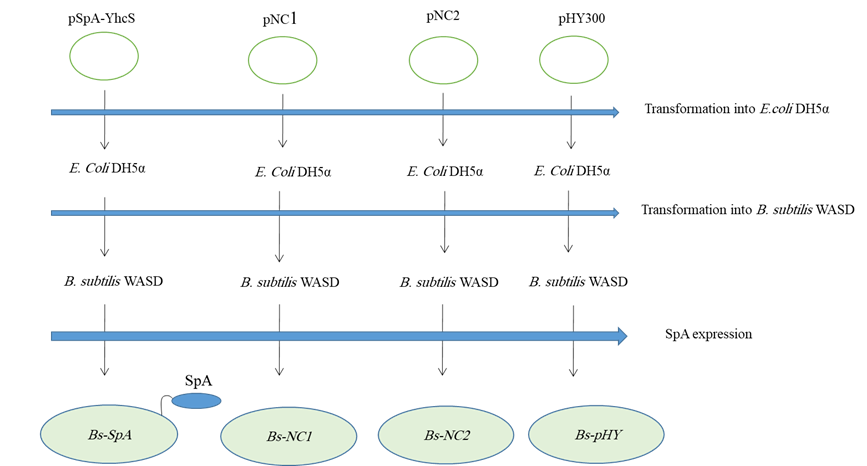


Figure S4. The process of making constructs and their transformation.
